# Rim Pathway-Mediated Alterations in the Fungal Cell Wall Influence Immune Recognition and Inflammation

**DOI:** 10.1128/mBio.02290-16

**Published:** 2017-01-31

**Authors:** Kyla S. Ost, Shannon K. Esher, Chrissy M. Leopold Wager, Louise Walker, Jeanette Wagener, Carol Munro, Floyd L. Wormley, J. Andrew Alspaugh

**Affiliations:** a Departments of Medicine/Molecular Genetics and Microbiology, Duke University School of Medicine, Durham, North Carolina, USA; b Department of Biology, the University of Texas at San Antonio, San Antonio, Texas, USA; c School of Medical Sciences, University of Aberdeen, Institute of Medical Sciences, Foresterhill, Aberdeen, United Kingdom; University of Texas Health Science Center

## Abstract

Compared to other fungal pathogens, *Cryptococcus neoformans* is particularly adept at avoiding detection by innate immune cells. To explore fungal cellular features involved in immune avoidance, we characterized cell surface changes of the *C. neoformans rim101*Δ mutant, a strain that fails to organize and shield immunogenic epitopes from host detection. These cell surface changes are associated with an exaggerated, detrimental inflammatory response in mouse models of infection. We determined that the disorganized strain *rim101*Δ cell wall increases macrophage detection in a contact-dependent manner. Using biochemical and microscopy methods, we demonstrated that the *rim101*Δ strain shows a modest increase in the levels of both cell wall chitin and chitosan but that it shows a more dramatic increase in chito-oligomer exposure, as measured by wheat germ agglutinin staining. We also created a series of mutants with various levels of cell wall wheat germ agglutinin staining, and we demonstrated that the staining intensity correlates with the degree of macrophage activation in response to each strain. To explore the host receptors responsible for recognizing the *rim101*Δ mutant, we determined that both the MyD88 and CARD9 innate immune signaling proteins are involved. Finally, we characterized the immune response to the *rim101*Δ mutant *in vivo*, documenting a dramatic and sustained increase in Th1 and Th17 cytokine responses. These results suggest that the Rim101 transcription factor actively regulates the *C. neoformans* cell wall to prevent the exposure of immune stimulatory molecules within the host. These studies further explored the ways in which immune cells detect *C. neoformans* and other fungal pathogens by mechanisms that include sensing N-acetylglucosamine-containing structures, such as chitin and chitosan.

## IMPORTANCE

Infectious microorganisms have developed many ways to avoid recognition by the host immune system. For example, pathogenic fungi alter their cell surfaces to mask immunogenic epitopes. We have created a fungal strain with a targeted mutation in a pH response pathway that is unable to properly organize its cell wall, resulting in a dramatic immune reaction during infection. This mutant cell wall is defective in hiding important cell wall components, such as the chito-oligomers chitin and chitosan. By creating a series of cell wall mutants, we demonstrated that the degree of chito-oligomer exposure correlates with the intensity of innate immune cell activation. This activation requires a combination of host receptors to recognize and respond to these infecting microorganisms. Therefore, these experiments explored host-pathogen interactions that determine the degree of the subsequent inflammatory response and the likely outcome of infection.

## INTRODUCTION

Over the last several decades, the increased use of immunosuppressive drugs and the HIV/AIDS pandemic have greatly expanded the population of people who are susceptible to disseminated fungal infections. The opportunistic fungal pathogen *Cryptococcus neoformans* has emerged as a particularly deadly pathogen, causing over 300,000 deaths each year, primarily among those suffering from HIV/AIDS (1, 2). *C. neoformans* first colonizes the lungs, where it can disseminate to the central nervous system to cause life-threatening fungal meningitis, which is universally fatal without treatment (1). The initial interactions between *C. neoformans* and the innate immune cells in the lung elicit either a robust, protective immune response or a weak, nonprotective response. This infection can also lead to an overexuberant pattern of immune activation resulting in excessive host damage that can be fatal (3). Understanding this initial host-microbe interaction will allow us to better define what constitutes a beneficial immune response to this pathogen.

*C. neoformans* has a highly dynamic cell surface that changes in composition and architecture during infection. Some of these changes include alterations in the cell wall carbohydrate composition and the attachment of a polysaccharide capsule (4–6). Alterations in the cell wall influence the interaction of *C. neoformans* with immune cells. The capsule, which is primarily composed of the polysaccharide glucuronoxylomannan (GXM), shields potentially immune-stimulatory molecules in the cell wall from detection. GXM also actively inhibits proinflammatory receptors and signaling in innate immune cells (7–11). While no detailed cell wall analysis has been performed during *C. neoformans* infection, increased levels of chitin and α-1,3-glucan in *C. neoformans* cells recovered from infected mice or from cells cultured in host-mimicking tissue culture media have been noted (4, 12). Additionally, the *C. neoformans* cell wall has been shown to thicken during infection (13). Within the host, during infection, *C. neoformans* produces Titan cells, representing a morphological state with a very thick cell wall and dense capsule (14, 15). The surface properties of Titan cells have recently been shown to guide the immune response to achieve a more favorable environment for *C. neoformans* survival (16). Compared to other fungal pathogens, *C. neoformans* has an increased amount of chitin and chitosan present in its cell wall and a corresponding decrease in the amounts of many α- and β-glucans and mannoproteins (17). Interestingly, relatively little is known about how the innate immune system recognizes distinct components of the *C. neoformans* cell surface and especially its cell wall.

Previously, members of our laboratory identified the *C. neoformans* Rim101 transcription factor as an important regulator of the adaptive cell surface changes that occur during infection. The *RIM101* gene is one of the most highly induced genes during human cryptococcosis, suggesting that it plays a central role in microbial survival *in vivo* (18). The *rim101*Δ mutant has a defect in capsule attachment, as well as an aberrant cell wall morphology (4, 19). Despite these cell surface defects, this mutant was unexpectedly hypervirulent in a mouse inhalation model of infection. Further investigation demonstrated that the *rim101*Δ strain induces a dramatic inflammatory response in the lungs of infected mice, leading to excessive host damage (4, 19).

The particular cell surface changes in the *rim101*Δ mutant that drive this increased inflammatory response were not immediately evident. During infection, and when cultured in host-mimicking tissue culture media, this mutant displays increased staining by wheat germ agglutinin (WGA), a reagent commonly used to detect and quantify chitin-derived fungal cell wall carbohydrates (4, 16, 20, 21). In previous studies, these have been defined in *C. neoformans* as chito-oligomers consisting of 3 to 20 residues of N-acetylglucosamine, which can include chitin and chitosan molecules (20). Chitin is a polymer composed of repeating subunits of β-1,4-*N*-acetyl glucosamine, serving as an important structural component of most fungal cell walls (22). Chitin makes up a particularly high proportion of the *C. neoformans* cell wall, and it is significantly more abundant in *C. neoformans* than in other important fungal pathogens such as *Candida albicans* (17, 23). *C. neoformans* cell walls also contain significantly more of the chitin-related carbohydrate chitosan than those of many other fungi (17, 24). Chitosan is a polymer of β-1,4-*N*-glucosamine and is synthesized by the deacetylation of chitin. A polymer that is at least 60% deacetylated is generally considered to be chitosan; however, these polymers can be heterogeneous, containing both *N*-acetylglucosamine and *N-*glucosamine residues (25, 26). Previous work has shown that immune recognition of chitin is a complex process and can result in both increased and decreased inflammatory responses, depending on the source and size of the chitin molecule (16, 24, 27). *C. neoformans* provides an excellent model to better understand the immune response to fungal cell wall chitin and chitosan.

In this study, we explored the Rim101-regulated cell surface properties that control innate immune detection. In addition to a previously described capsule attachment defect, we find that the *rim101*Δ mutant has a modest increase in total cell wall chitin and chitosan levels. However, the *rim101*Δ mutant also displays a previously unappreciated increase in chito-oligomer exposure, as demonstrated by staining with WGA. We show that these cell wall defects result in increased recognition by macrophages *in vitro* and that this response likely involves members of the Toll-like receptor (TLR) and C-type lectin receptor (CLR) families. We also demonstrate that the *rim101*Δ mutant induces a dramatic inflammatory response in the lungs of infected mice that persists throughout a prolonged infection. Our results demonstrate that a properly organized *C. neoformans* cell wall is not readily recognized by macrophages and that it is much less stimulatory than the cell walls from other common fungal pathogens (28–30). Active regulation of the *C. neoformans* cell wall architecture is important for evading immune detection. In particular, increased WGA staining is associated with an increase in *C. neoformans* recognition by the immune system, altering the balance between pathogen recognition/clearance and host recovery from immune activation.

## RESULTS

### The *rim101Δ* mutation increases macrophage activation.

**FIG 1:**
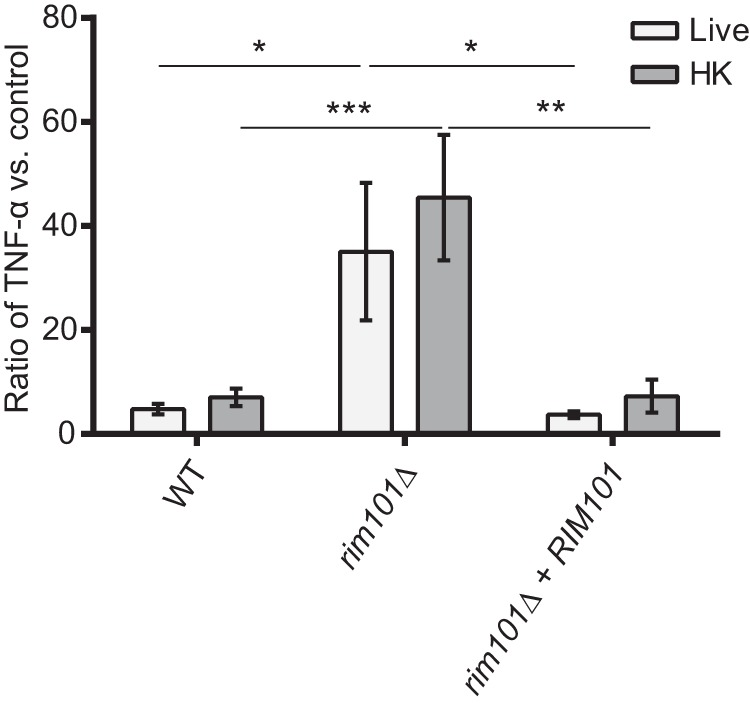
The *rim101*Δ mutant induces increased TNF-α secretion from macrophages. BMMs were coincubated with the indicated live or heat-killed (HK) strains at a multiplicity of infection (MOI) of 10:1 *C. neoformans*/BMM cells. Secreted TNF-α levels were quantified after 6 h of coincubation, and data are represented as a ratio of TNF-α levels (in picograms per milliliter) normalized to a control incubation with no added fungal cells. Primary data for TNF-α levels are listed in Table S1. Two-way analysis of variance (ANOVA) and Tukey’s multiple-comparison test were used to compare the means of results from three replicates for three independent experiments (*n* = 9). ***, *P* < 0.001; **, *P* < 0.01; *, *P* < 0.05. Error bars represent standard errors of the means.

We previously noted that the *rim101Δ* mutant induces a dramatic inflammatory response in the lungs of infected mice (4). During a *C. neoformans* infection, macrophages serve in the initial identification of and response to the pathogen. To determine whether macrophages would respond differently to the *rim101Δ* mutant, we quantified tumor necrosis factor alpha (TNF-α) secreted by bone marrow-derived macrophages (BMMs) that were cocultured with the mutant strain and compared the results to the levels seen with the wild-type (WT) and *rim101Δ + RIM101* reconstituted strains (Fig. 1). While this *in vitro* coculture system does not include all opsonins and other factors that may be present during a true infection, it does measure direct fungal activation of these immune cells. The *rim101*Δ strain was previously shown to induce significantly more TNF-α in the lungs of infected mice (4). This cytokine is also one marker of macrophage activation *in vitro*. Overall, we observed a TNF-α response to all *C. neoformans* strains that was much lower than previously published macrophage responses to other prominent fungal pathogens (30–33). In this assay, the *rim101Δ* mutant induced BMMs to secrete significantly more TNF-α than the WT. We observed a similar pattern of TNF-α secretion when the fungal cells were heat killed prior to coincubation with BMMs (Fig. 1). UV-killed fungi produced a similar response (data not shown). Therefore, the enhanced immune cell response to the *rim101Δ* strain does not appear to require fungal cell viability or metabolism.

**FIG 2:**
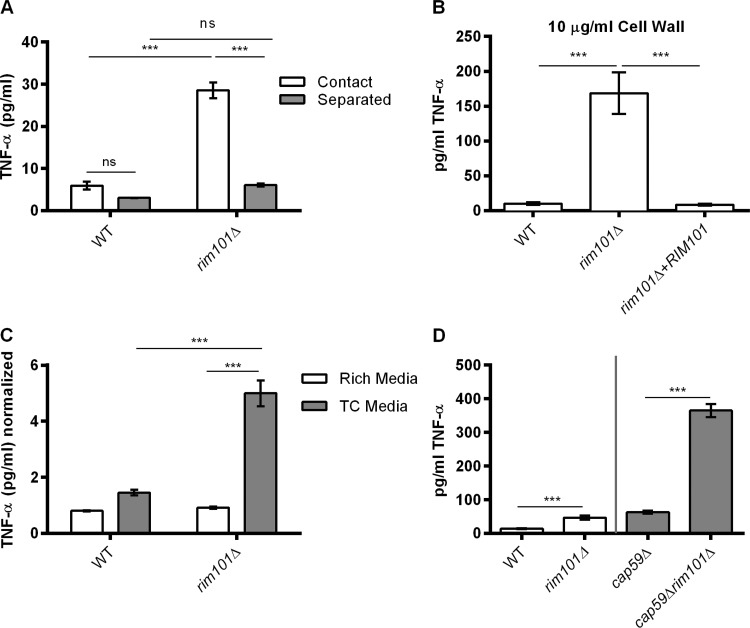
The *rim101*Δ mutant cell wall induces TNF-α secretion from BMMs in a contact-dependent and capsule-independent manner. (A) WT and *rim101*Δ cells were coincubated with BMMs at an MOI of 10:1 *C. neoformans*/BMM cells, either “in direct contact” or “separated” by a Transwell system. (*n* = 3 for each strain/condition.) (B) BMM TNF-α levels (in picograms per milliliter) after 6 h of incubation with 10 µg/ml purified cell wall material from the indicated fungal strains. Data represent means of results from three replicate samples for three independent cell wall preparations (*n* = 9 for each strain). (C) BMMs were coincubated for 6 h with UV-killed fungal cells that had been precultured in either YPD (rich) medium or tissue culture (TC), CO_2_-independent medium. TNF-α levels in the culture medium were normalized to a control incubation with no added fungal cells. Primary data for TNF-α levels are listed in Table S2. Data represent means of results from three replicate samples for two independent experiments (*n* = 6 for each strain/condition). (D) BMMs were coincubated with the indicated strains for 6 h. The gray line separates strains normalized by cell number (WT and *rim101*Δ strains) and by weight (*cap*59Δ strain or *cap*59Δ *rim101*Δ strain). Data represent means of results from three replicate samples (*n* = 9). Two-way (A) and one-way (B, C, and D) ANOVA and Tukey’s multiple-comparison test were used to compare means. ***, *P* < 0.001; **, *P* < 0.01; *, *P* < 0.05. Error bars represent standard errors of the means.

These data demonstrate that certain static properties of the *rim101*Δ mutant cells are immune stimulatory. We utilized this *in vitro* measure of macrophage stimulation to further analyze innate immune cell interaction with the *rim101*Δ mutant. To determine whether the response to this mutant was contact dependent, we used a 3-µm-pore-size Transwell system to separate fungal and BMM cells in a coculture assay (Fig. 2A). Without Transwell separation, we again observed an increased TNF-α response to the *rim101*Δ mutant compared to the response to the WT strain. With Transwell separation, however, we observed no significant difference in the TNF-α responses to the various strains. This result suggests that the observed enhanced macrophage response requires direct contact with the *rim101*Δ strain and also suggests that the response to this mutant is not driven by a secreted immune stimulatory molecule.

Rim101 is a central regulator of the cell wall during infection, and mutations disrupting Rim101 function result in an altered cell surface (4). To determine whether the *rim101*Δ mutant cell wall could independently induce increased BMM activation, we purified cell wall material from the *C. neoformans* strains after growth in tissue culture medium was performed and we treated BMMs with 10 µg/ml of the cell wall extracts. Similarly to whole cells, cell wall material isolated from the *rim101*Δ mutant induced significantly more TNF-α from BMMs than the cell wall material isolated from either the WT or the reconstituted strain (Fig. 2B). Examined microscopically, the isolated cell wall material was composed of empty cell-shaped particles (data not shown), indicating that architecture of the cell wall may be largely maintained in the cell wall extracts. Together, these data suggest that macrophages recognize *rim101*Δ *C. neoformans* more readily than WT *C. neoformans*, which is likely due to changes in the composition or architecture of the *rim101*Δ cell wall.

The *rim101*Δ mutant cell surface defects manifest only when the strain is cultured under host-mimicking conditions (tissue culture medium), and the mutant has a normal-appearing cell wall when cultured in rich medium (4). We determined whether the BMM response to the *rim101*Δ mutant would also be dependent on the growth medium. First, we precultured WT and *rim101*Δ mutant cells in rich medium (yeast extract-peptone-dextrose [YPD]) or tissue culture medium. In order to prevent active cell wall remodeling during coincubation with BMMs, we killed the fungal cells using UV irradiation prior to coculture. Cultured under tissue culture conditions, the *rim101*Δ mutant again induced more TNF-α secretion than the WT cells (Fig. 2C). In contrast, these strains induced similar levels of TNF-α after the fungi were precultured in rich medium. This demonstrates that the *rim101*Δ mutant is stimulatory only when cultured under conditions that induce cell surface alterations.

### The *rim101*Δ mutation increases immune recognition of the acapsular *cap59Δ* mutant.

*C. neoformans* polysaccharide capsule components actively suppress innate immune cell activation, and they also serve to shield the more immunogenic cell wall from recognition (7, 32). As such, we considered that the previously reported *rim101*Δ capsule defect could potentially explain the increased immune recognition of these mutant strains. To determine the role of the capsule in the recognition of the *rim101*Δ strain, we compared the BMM responses to strain *cap59*Δ, the acapsular mutant, and the *rim101*Δ *cap59*Δ double mutant. Due to cell aggregation in the *cap59*Δ background, we normalized the cell concentrations based on weight instead of cell number. Overall, we observed an increased TNF-α response to both acapsular strains (the *cap59*Δ and *rim101*Δ *cap59*Δ mutants) compared to the WT strain (Fig. 2D), though the precision of the comparisons was limited due to differing cell normalization methods. We also noted that the *cap59*Δ *rim101*Δ double mutant induced significantly more TNF-α production than the *cap59*Δ single mutant (Fig. 2D). These data indicate that the *rim101*Δ mutant cell wall changes increase immune recognition by another mechanism in addition to the effect due to loss of surface capsule.

### Analysis of cell wall carbohydrate content and organization in the *rim101*Δ mutant cell wall.

We previously demonstrated that host-mimicking conditions *in vitro* induced the *rim101*Δ mutant to produce a thick, disordered cell wall, a phenotype that was absent when cells were cultured in rich media (4). The *rim101*Δ mutant cell wall demonstrated a dramatic increase in cell surface staining with wheat germ agglutinin (WGA), a lectin that binds chito-oligomers (4). This result suggested the presence of a very different cell wall structure in this strain.

**FIG 3:**
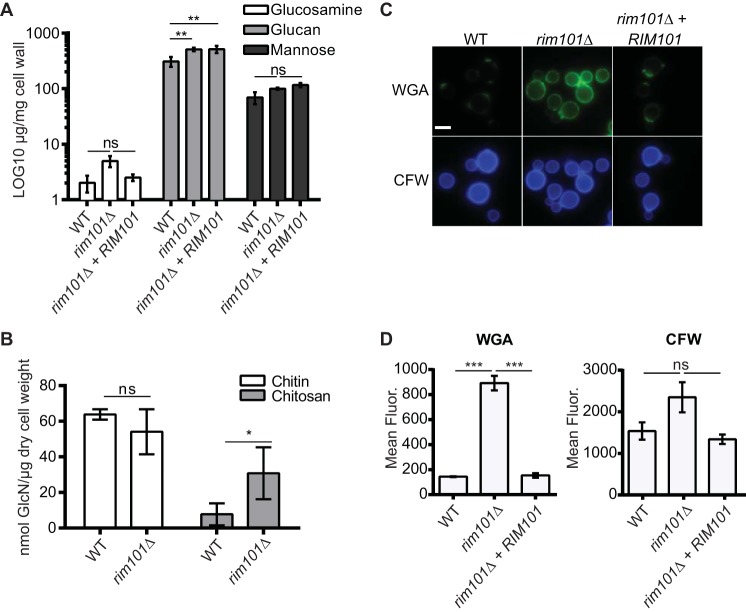
The *rim101*Δ mutant has increased chito-oligomer exposure. (A) The cell walls were isolated from the WT, *rim101*Δ mutant, and *rim101Δ + RIM101* reconstituted strains after incubation for 18 h in CO_2_-independent medium. Glucosamine, glucan, and mannose levels were quantified in the isolated cell walls using high-performance anion-exchange chromatography with pulse amperometric detection (HPAEC-PAD). Data represent means of results from three independent cell wall preparations (*n* = 3 for each strain). (B) The WT and *rim101*Δ mutant were incubated for 18 h in CO_2_-independent medium, and cell walls were isolated. Chitin and chitosan levels were quantified for each strain using a modified 3-methyl-2-benzothiazolinone hydrazone hydrochloride (MBTH) colorimetric assay. Data represent means of results from three independent cell wall preparations (*n* = 3 for each strain). (C) *C. neoformans* strains were incubated for 18 h in CO_2_-independent medium and stained with fluorescein isothiocyanate (FITC)-conjugated wheat germ agglutinin (WGA) or calcofluor white (CFW). Staining intensity was assessed using epifluorescence microscopy with identical exposures for all images. (D) Fluorescence levels for 100 individual cells (represented in panel C) were quantified using ImageJ (Fiji). One-way ANOVA and Tukey’s multiple-comparison test were used to compare means to the WT values. Means represent the fluorescent (Fluor.) intensity levels from three independent experiments (*n* = 3). ***, *P* < 0.001; **, *P* < 0.01; *, *P* < 0.05; ns, not significant. Error bars represent standard errors of the means.

To better define the actual composition of the *rim101*Δ mutant cell wall compared to that of the WT, we biochemically quantified the levels of three major cell wall carbohydrates that comprise a typical fungal cell wall: glucosamine, glucan, and mannose. After culturing the strains in host-like tissue culture media, the cell walls were extracted and hydrolyzed into their individual sugar components. This technique deacetylates the chitin to chitosan and therefore measures chitin and chitosan together as glucosamine. The levels of glucosamine, glucan, and mannose in these cell wall fractions were quantified using high-performance liquid chromatography (HPLC) (Fig. 3A). We found that the levels of mannose, which is found on cell wall mannoproteins, were not significantly different between the WT, *rim101*Δ, and *rim101*Δ + *RIM101* strains. Changes in total glucose/glucan content were also not observed between the *rim101*Δ and *rim101*Δ + *RIM101* strains. The WT strain had slightly less glucose/glucan than the *rim101*Δ and reconstituted strains. As the WT and *rim101*Δ + *RIM101* strains induce similar macrophage responses, the slightly altered glucan content likely does not contribute to the increased immune response to the *rim101*Δ mutant. The mannose-rich capsule did not appear to contaminate our analysis, as the acapsular *cap59*Δ strain had mannose levels comparable to those seen with the WT cells (data not shown). Despite the relatively low level of glucosamine, we observed a trending though statistically insignificant increase in the level of glucosamine in the *rim101*Δ mutant compared to both the WT strain (*P* = 0.998) and the *rim101*Δ + *RIM101* reconstituted strain (*P* = 0.999). Overall, by this method, the total relative levels of glucosamine, glucan, and mannose in the *rim101*Δ mutant cell wall were not dramatically different.

To differentiate between alterations in the chitin and chitosan levels in the *rim101*Δ mutant, we utilized a recently developed biochemical assay to quantify these components separately (reference 23 and Jennifer Lodge, personal communication). We found no significant difference in chitin levels between the *rim101*Δ mutant and WT strains, but we did observe a statistically significant increase in chitosan levels in the mutant strain (Fig. 3B). Our results also demonstrate that the growth medium likely alters chitin and chitosan levels in *C. neoformans*, as cells cultured in a rich medium have been shown to have chitosan/chitin ratios that are higher than what we observed here from cells cultured in tissue culture medium (23).

We previously observed a dramatic increase in the staining intensity of the *rim101*Δ mutant cell wall with the chitin-binding lectin WGA when this strain was incubated under host-mimicking conditions such as those using tissue culture medium. This observation suggested that the *rim101*Δ mutant cell wall might contain significantly higher levels of chito-oligomers or other N-acetylglucosamine-containing molecules (4, 34). However, this result is not consistent with the relatively small increases in relative chitin/chitosan levels observed in the HPLC analysis and biochemical chitin/chitosan quantification. To explore the discrepancy between the WGA staining and HPLC analysis, we analyzed *rim101*Δ mutant cell wall chitin levels using a different fluorescent stain, calcofluor white (CFW). CFW is a small (917-Da) fluorescent molecule that also binds chitin and chitosan, in addition to cellulose (35). We compared the mean CFW fluorescent levels of WT, *rim101*Δ, and *rim101*Δ + *RIM101* reconstituted strains that were cultured in tissue culture medium, the condition under which we observed increased WGA binding (Fig. 3C). We also stained the cells with WGA. Similarly to our previous study, we found that WGA cell wall fluorescence was 6-fold higher for the *rim101*Δ mutant than for the WT and *rim101*Δ + *RIM101* reconstituted strains. For the same cells, the CFW fluorescence was only 1.5-fold higher in the *rim101*Δ mutant cells than in the WT and *rim101*Δ + *RIM101* strains. This observation is more consistent with the biochemical measurement of total chitin and chitosan levels in these strains (Fig. 3B). CFW also displayed a staining pattern different from that of WGA, with CFW staining the entire cell wall whereas WGA brightly stained only the bud necks and bud scars on the WT and *rim101*Δ + *RIM101* strains, as well as showing punctate staining throughout the *rim101*Δ cell surface. Chitin and chitosan make up the basal, structural layer of the fungal cell wall, and these carbohydrates are present in higher levels at bud necks and bud scars of yeast cells (22). This suggests that WGA, which is a 38-kDa protein dimer (34), primarily binds exposed chito-oligomers, while the much smaller CFW molecule has access to total chito-oligomers present in the fungal cell wall (36, 37). Together, these data suggest that the *rim101*Δ cell wall has a modest increase in total chitin/chitosan content but displays an altered pattern of organization resulting in a more dramatic increase in chito-oligomer exposure.

### Alteration in chitin and chitosan synthesis and exposure increases recognition by BMMs.

*C. neoformans* possesses eight chitin synthase genes (*CHS1* to *CHS8* [*CHS1-8*]) that may be involved in distinct temporal, morphological, or site-specific aspects of chitin biosynthesis. Similarly to the case in other fungi, this apparent gene family expansion results in potential functional redundancy for synthesizing the fundamental components of such an important biological structure as the cell wall. Possession of multiple chitin-modifying genes also suggests the potential for precise, condition-specific modification of the molecular structure of chitin and chitosan (23, 38, 39). However, despite some degree of functional overlap, previous studies demonstrated that mutations disrupting single *CHS* genes result in measurable alterations in chitin and chitosan levels. For example, the *chs3*Δ mutant cannot produce chitosan, and the *chs5*Δ mutant has a marked decrease in total levels of cell wall chitin (23).

**FIG 4:**
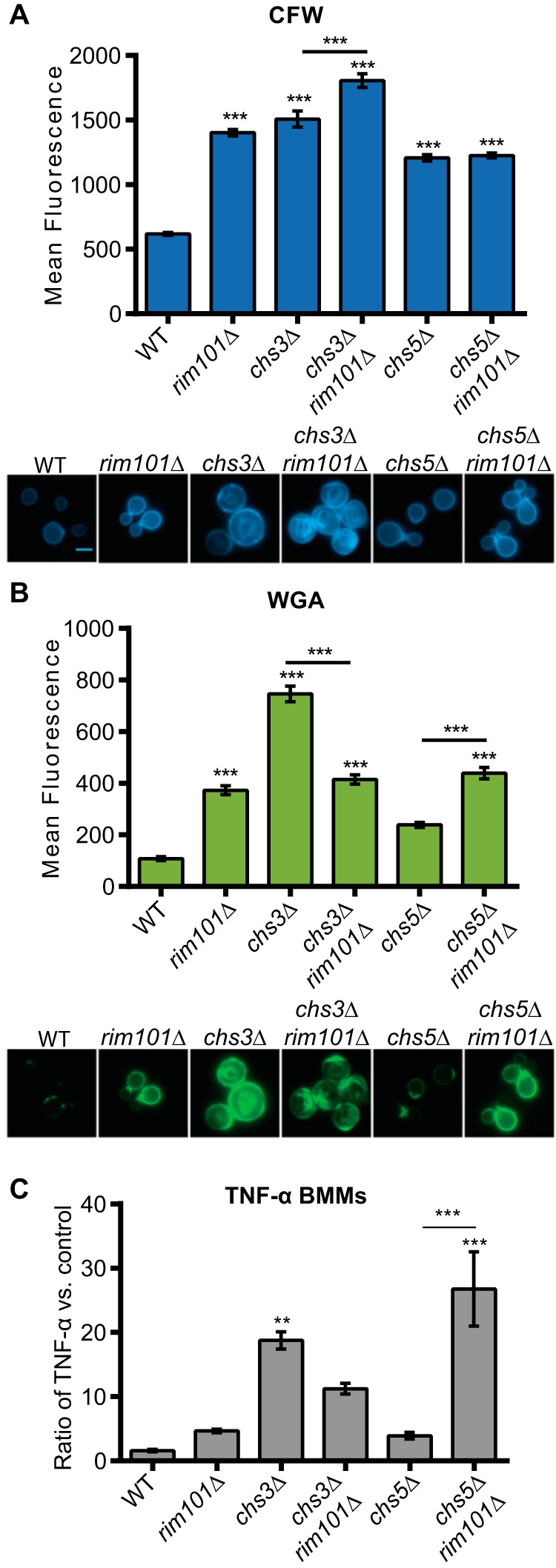
Fungal cell WGA staining intensity correlates with levels of induced TNF-α secretion from BMMs. (A and B) *C. neoformans* strains were stained with CFW (A) or FITC-WGA (B) after incubation in tissue culture medium at 37°C for 18 h. Fluorescence levels for 100 individual cells were quantified using ImageJ (Fiji) (upper panels) (*n* = 100). Representative fluorescent microscopic images for each strain (at a magnification of ×600) are demonstrated in the lower panels. (C) *C. neoformans* strains were coincubated with BMMs at a multiplicity of infection (MOI) of 10:1 *C. neoformans*/BMM cells. TNF-α levels in the culture medium were normalized to a control incubation with no added fungal cells. One-way ANOVA and Tukey’s multiple-comparison test were used to compare the means of results from three independent experimental samples. ***, *P* < 0.001; **, *P* < 0.01; *, *P* < 0.05. Error bars represent standard errors of the means.

We hypothesized that increased chitin and chitosan levels in the *rim101*Δ mutant cell wall were responsible, at least partially, for its increased immune recognition. Therefore, we sought to decrease both chitin and chitosan levels by disrupting the *CHS3* (chitosan) and *CHS5* (chitin) biosynthetic genes in the *rim101*Δ mutant background. In previously published data from experiments using biochemical assays and a rich culture medium, the same *chs* mutants displayed alterations in either chitin or chitosan content even though the total chitin/chitosan levels were not dramatically changed. However, our culturing of the resulting *chs* mutant strains in tissue culture medium induced unexpected effects on cell wall staining of these chito-oligomers. Interestingly, neither the *chs3*Δ nor the *chs5*Δ mutation decreased CFW or WGA staining in the *rim101*Δ strain. Additionally, the *chs3*Δ and *chs5*Δ mutations in the WT background resulted in increased CFW and WGA staining (Fig. 4A and B). Together, these data suggest that *chs3*Δ and *chs5*Δ mutations have differing effects on cell wall chito-oligomer levels (CFW staining) and chito-oligomer exposure (WGA staining) that depend on the background of the mutant strain and the growth conditions.

By disrupting *CHS3* and *CHS5* in the *rim101*Δ mutant, we inadvertently created a series of strains with various levels of CFW and WGA staining. We used these strains as tools to further probe the association between macrophage activation and CFW/WGA staining intensities. Consistent with our previous experiments, we observed that the *rim101*Δ strain induced more TNF-α secretion by BMMs than the WT strain, although this increase did not reach statistical significance in this experiment. We also noted that the *chs3*Δ, *chs3*Δ *rim101*Δ, and *chs5*Δ *rim101*Δ mutants induced even more TNF-α secretion than the *rim101*Δ strain, while the *chs5*Δ mutation alone had no significant effect of the TNF-α levels (Fig. 4C). Importantly, the level of WGA staining of each strain correlated with the level of BMM TNF-α secretion.

The *chs3* mutation did result in an alteration of cell size after incubation in this tissue culture medium, with increases in average cell area compared to the wild type of 3.8-fold for the *chs3*Δ mutant and 2.6-fold for the *chs3*Δ *rim101*Δ double mutant. However, this cellular change is insufficient to account for the larger measured increases in WGA intensity for these cells (Fig. 4C). In contrast, increased CFW staining was not associated with increased BMM TNF-α secretion in each case (Fig. 4). The *chs5*Δ *rim101*Δ double mutant was somewhat of an exception to this pattern, as it induced significantly more TNF-α secretion than the other tested strains. Together, these data suggest that increased chito-oligomer exposure, but not necessarily the total cell wall chitin or chitosan level, is closely associated with increased macrophage activation.

### Macrophage activation *in vitro* does not require the inflammasome.

Previous studies have demonstrated that purified chitosan activates the inflammasome and induces inflammatory cytokine secretion (40, 41). As such, we considered that elevated chitosan levels in the *rim101*Δ mutant cell wall might increase inflammasome activation. Two signals are required for inflammasome activation: an initial Toll-like receptor (TLR)- or C-type lectin receptor (CLR)-activating signal and a subsequent stimulus that activates one of the NLRP inflammasomes. Activation induces the production, processing, and secretion of the inflammatory cytokines interleukin-33 (IL-33), IL-18, and IL-1β. We treated both lipopolysaccharide (LPS)-primed (TLR-stimulated) and unprimed BMMs with the WT, *rim101*Δ, *rim101*Δ + *RIM101*, *cap59Δ*, and *rim101*Δ *cap59*Δ strains. We then quantified the level of secreted IL-1β. IL-1β levels were not detected above baseline for any strain (Fig. S1A and B). As a positive control, we also treated BMMs with purified chitosan, and we observed elevated IL-1β secretion from LPS-primed BMMs (Fig. S1C). Therefore, in this *in vitro* assay, the *rim101*Δ mutation does not appear to have induced macrophage activation through inflammasome-mediated signaling recognizing its increased cell wall chitosan content or exposure.

10.1128/mBio.02290-16.1 FIG S1  The *rim101*Δ mutation does not induce measurable IL-1β secretion (A and B). BMMs were primed with or without 100 ng/ml UltraPure LPS for 3 h. BMMs were coincubated with the indicated *C. neoformans* strains at an MOI of 10 *C. neoformans* fungal cells to 1 BMM. *cap59*Δ and *rim101*Δ *cap59*Δ cells were normalized to 2 mg cells/ml of media (C) LPS-primed and unprimed BMMs were treated with 0.1 mg/ml chitosan for 6 h. Secreted IL-1β levels (in picograms per milliliter) were quantified by ELISA (BioLegend) after 6 h of coincubation. Two-way or one-way ANOVA and Tukey’s multiple-comparison test were used to compare means of results of three replicates for each strain (*n* = 3). ***, *P* < 0.001; **, *P* < 0.01; *, *P* < 0.05. Error bars represent standard errors of the means. Download FIG S1, EPS file, 0.8 MB. Copyright © 2017 Ost et al. 2017 Ost et al. This content is distributed under the terms of the Creative Commons Attribution 4.0 International license.

### *In vitro* response to the *rim101*Δ strain involves MyD88 and CARD9.

Macrophages recognize and respond to fungi through the activation of multiple pattern recognition receptors, or PRRs. Of these receptors, members of the Toll-like receptor (TLR) and the C-type lectin receptor (CLR) families have been shown to be responsible for recognizing fungal cell wall carbohydrates and mannoproteins (42). To determine whether members of the TLR or CLR families are responsible for the increased recognition of the *rim101*Δ mutant strain, we utilized BMMs from mice lacking key downstream signaling adaptor proteins required for TLR and CLR activity. Most TLRs require the MyD88 adaptor protein to activate cytokine production, while the CLRs require CARD9 to propagate their downstream stimulatory signals (42).

**FIG 5:**
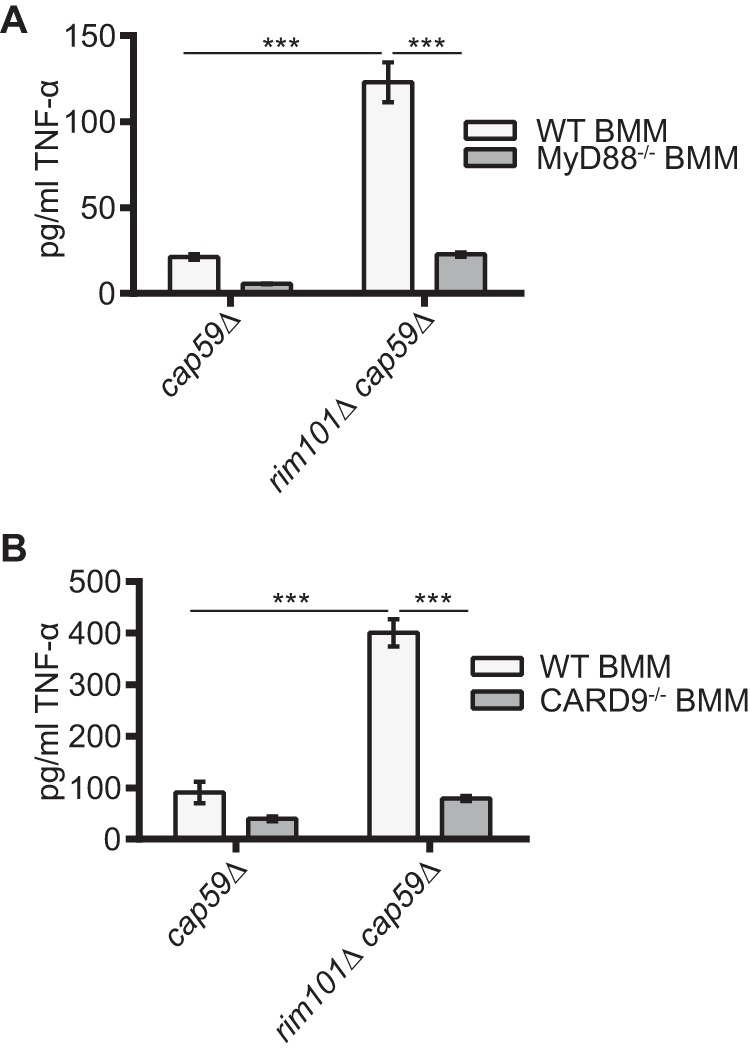
MyD88 and CARD9 contribute to the TNF-α response to the *rim101*Δ strain. BMMs were harvested from WT (A and B) and MyD88^−/−^ mutant (A) or CARD9^−/−^ mutant (B) mice in the C57BL-6 background. These cells were coincubated with 2 mg/ml of the *cap59*Δ strain or the *rim101*Δ *cap59*Δ strain. TNF-α levels (in picograms per milliliter) in the culture medium were quantified after 6 h coincubation. Two-way ANOVA and Tukey’s multiple-comparison test were used to compare the means of results from three replicate samples (*n* = 3). ***, *P* < 0.001; **, *P* < 0.01; *, *P* < 0.05. Error bars represent standard errors of the means.

We coincubated *C. neoformans* strains with BMMs derived from MyD88^−/−^ or CARD9^−/−^ mutant mice, and we compared their TNF-α responses to those seen with WT BMMs. To better visualize subtle alterations in the TNF-α response to the *rim101*Δ mutant, we utilized the acapsular *cap59*Δ mutant background to amplify the TNF-α response (Fig. 5). Although the responses to *cap59*Δ *C. neoformans* cells were not significantly altered in the MyD88^−/−^ and CARD9^−/−^ BMMs, these mutant macrophages failed to induce TNF-α production in response to the *rim101*Δ *cap59*Δ strain (Fig. 5). These results demonstrate that the increased recognition of the *rim101*Δ *cap*59Δ strain by macrophages requires both MyD88 and CARD9, suggesting that one or more members of the TLR and CLR families are involved.

### The *rim101*Δ mutant induces a global increase in inflammatory cytokine production in the lungs of infected mice.

**FIG 6:**
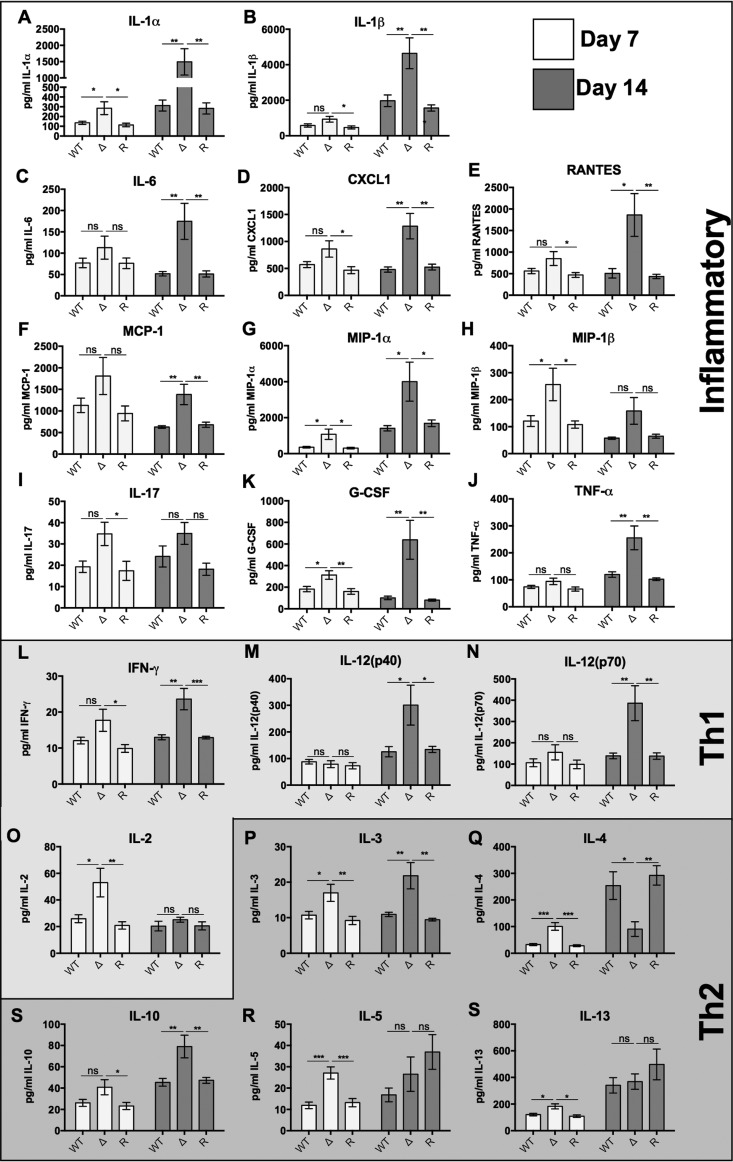
The *rim101*Δ strain elicits an inflammatory and Th1 cytokine response in infected lungs. The WT, *rim101*Δ mutant, and *rim101Δ + RIM101* reconstituted strains were inoculated (10^4^ fungal cells) into BALB/c mice by inhalation. At day 7 and day 14 postinfection, the indicated cytokines were quantified from whole-lung digestions. 4 mice were inoculated for each strain. Data represent results of 2 independent experiments (8 mice in total). Primary data for cytokine levels are listed in Table S3. One-way ANOVA and Tukey’s multiple comparison tests were used to compare means of results to the WT infection data for each day. G-CSF, granulocyte colony-stimulating factor. ***, *P* < 0.001; **, *P* < 0.01; *, *P* < 0.05. Error bars represent standard errors of the means.

To better characterize the immune response to the *rim101*Δ mutant, we intranasally inoculated BALB/c mice with 10^4^ cells of each strain and analyzed the levels of 18 cytokines in infected lungs at day 7 and day 14 postinfection (Fig. 6; Table S3). In the *rim101*Δ mutant-infected lungs, there were increased levels of several inflammatory cytokines measured at day 7 and a global increase in the levels of almost all cytokines measured at day 14 compared to the levels seen with the WT and *rim101*Δ + *RIM101* mutant infections. Several of the most highly upregulated cytokines, including the neutrophil chemoattractant CXCL1 (Fig. 6D) and the inflammatory cytokines IL-1α, IL-1β, TNF-α, IL-6, monocyte chemoattractant protein 1 (MCP-1), macrophage inflammatory protein 1 alpha (MIP-1α), and MIP-1β (Fig. 6), are known to induce inflammatory cell infiltration. Several Th1-, Th2-, and Th17 (inflammatory)-driving cytokines were upregulated by the *rim101*Δ mutant, making it difficult to determine the specific type of adaptive immune response induced by this strain. Notably, the only cytokine whose level was significantly reduced in the *rim101*Δ-infected lungs at day 14 was the Th2-associated cytokine IL-4 (Fig. 6Q). In contrast, the levels of key Th1 and Th17 (inflammatory) polarizing cytokines, such as IFN-γ and IL-17A, were significantly higher or trended higher at day 14 (Fig. 6). Altered cytokine profiles were not due to increased proliferation of the *rim101*Δ mutant, as similar numbers of viable *rim101*Δ mutant and WT cells were recovered from the lung and the brain at day 7 (lung only) and day 14 (lung and brain) postinfection (Fig. S2). Overall, the *rim101*Δ mutant induced a dramatic immune response in the lungs of infected mice, characterized by a global increase in cytokine secretion.

10.1128/mBio.02290-16.4 TABLE S1  TNF-α levels (quantified in picograms per milliliter) for Fig. 1. Download TABLE S1, DOCX file, 0.01 MB. Copyright © 2017 Ost et al. 2017 Ost et al. This content is distributed under the terms of the Creative Commons Attribution 4.0 International license.

10.1128/mBio.02290-16.5 TABLE S2  TNF-α levels (quantified in picograms per milliliter) for Fig. 2C. Data represent the BMM TNF-α response to *C. neoformans* cultured in rich media versus tissue culture media. Download TABLE S2, DOCX file, 0.01 MB. Copyright © 2017 Ost et al. 2017 Ost et al. This content is distributed under the terms of the Creative Commons Attribution 4.0 International license.

10.1128/mBio.02290-16.2 TABLE S3  All cytokines quantified. Download TABLE S3, DOCX file, 0.02 MB. Copyright © 2017 Ost et al. 2017 Ost et al. This content is distributed under the terms of the Creative Commons Attribution 4.0 International license.

10.1128/mBio.02290-16.3 FIG S2  The *rim101*Δ mutant proliferates at a rate similar to that of the WT strain during infection. Fungal CFU counts were determined from whole-lung (A and B) or whole-brain (C) homogenates. 4 BALB/c mice were intranasally inoculated with 10^4^ cells for each strain. Data represent results of 2 independent experiments (8 mice in total). One-way ANOVA and Tukey’s multiple-comparison test were used to compare means. ***, *P* < 0.001; **, *P* < 0.01; *, *P* < 0.05. Error bars represent standard errors of the means. Download FIG S2, EPS file, 1 MB. Copyright © 2017 Ost et al. 2017 Ost et al. This content is distributed under the terms of the Creative Commons Attribution 4.0 International license.

## DISCUSSION

**FIG 7:**
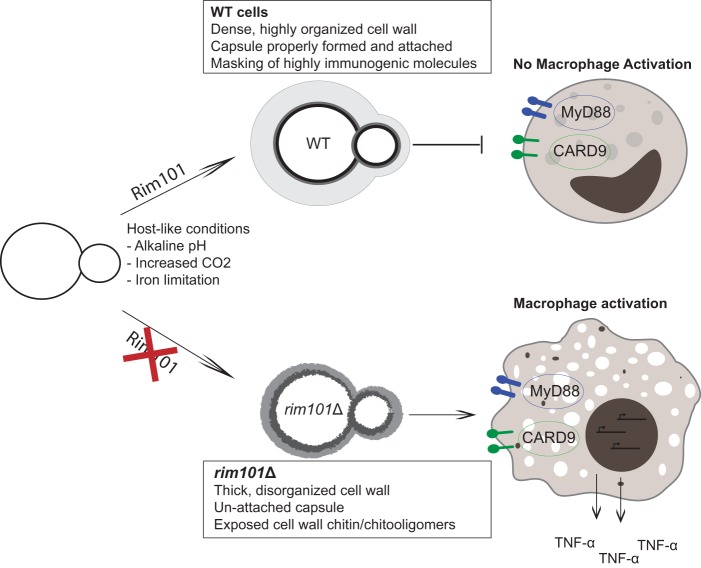
Model for the immune stimulatory mechanism of the *rim101*Δ mutant. When *C. neoformans* enters the host, it senses host environmental cues (elevated pH, increased CO_2_, high temperature) and uses the Rim101 signaling pathway to adaptively regulate various cellular structures. These adaptations include the organization of its cell wall and the production/attachment of its polysaccharide capsule. Proper cell surface regulation prevents recognition by innate immune receptors. Without Rim101 regulation, the cell wall becomes highly disorganized, resulting in increased chitosan levels, a dramatic increase in chito-oligomer exposure, and impaired capsule attachment. These cell surface defects allow enhanced detection by CARD9- and MyD88-dependent innate immune receptors, leading to increased inflammatory cytokine secretion by host immune cells.

Like many fungal pathogens, *C. neoformans* has a highly dynamic cell surface that it actively modifies during infection, thereby shaping its interaction with the immune system. Resulting immune responses can range from nonprotective and weak, in the case of cryptococcal meningitis, to overexuberant and damaging. Damaging immune responses to *C. neoformans* infection have become significant issues for people recovering from immune suppression, especially patients with late-stage HIV infection as they initiate antiretroviral therapy. This condition is known as immune reconstitution inflammatory syndrome, or IRIS, and it results in mortality rates similar to those encountered in cases of untreated *C. neoformans* meningitis (3). Typical infection models of cryptococcosis explore infection in highly susceptible hosts with nonprotective, weak immune responses. Few models explore the damaging immune responses that can also occur during these infections in humans. Therefore, little is known about the *C. neoformans* cellular attributes that drive highly active inflammatory responses, including microbial features that are typically hidden during infection with natural fungal isolates. *C. neoformans* cells induce a much lower innate immune response than other prominent fungal pathogens. Therefore, *C. neoformans*-driven inflammatory responses are difficult to analyze *in vitro*. The *rim101*Δ mutant was previously shown to induce a detrimental inflammatory response and an increased death rate in two separate mouse strains (4, 19). Alterations in the cell wall structure and composition, and the resulting defect in surface capsule, were hypothesized to increase immune recognition and drive the inflammatory response. In this study, as modeled in Fig. 7, we demonstrated that the *rim101*Δ mutant has increased chito-oligomer exposure on its cell surface in response to host-like conditions, without a major change in total cell wall carbohydrate content. This cell wall alteration, in addition to its decreased capsule, leads to increased recognition by macrophages *in vitro* and increased inflammation *in vivo* (Fig. 7).

Rim101 limits *C. neoformans* chitin exposure and chitosan levels under host-mimicking conditions and correspondingly suppresses recognition by innate immune cells. While other *rim101*Δ cell surface attributes undoubtedly contribute to immune recognition, our data strongly associate chito-oligomer exposure with immune stimulation by *C. neoformans*. Exposure of these carbohydrate molecules also appeared to be more important than total chitin or chitosan levels, which suggests that cell wall components are typically detected by immune cells primarily at the outer, exposed cell surface, perhaps emphasizing why fungi tend to place more immunogenic molecules deeply within the cell wall structure.

### Cell surface alterations that contribute to *rim101*Δ mutant immune activation.

Recent studies have demonstrated that chitin-derived structures are important in *C. neoformans* capsule formation and immune recognition. When WGA was added to cryptococcal cultures, the capsule polysaccharide failed to form, likely due to altered capsule gene expression and trafficking of capsular polysaccharide to the cell surface (21). Additionally, blocking surface chito-oligomers with WGA resulted in defective phagocytosis by macrophages (21). These results are consistent with our findings in which chito-oligomer exposure enhanced macrophage activation *in vitro* and *in vivo*.

The role of chitin in the immune response to fungi is complex. Unlike β-1,3-glucan, which is usually proinflammatory, chitin has been shown to both stimulate and inhibit immune responses, depending on the source and size of the chitin molecule. The reported type of immune response to chitin can range from an allergic Th2 response (24, 43) to a proinflammatory Th1 response (44–46) to an anti-inflammatory response (24, 47, 48). Several recent studies have begun to reveal the mechanisms behind the observed differing immune responses to fungal chitin. Wagener et al. found that initial interactions between BMMs and purified chitin from several fungal pathogens, including *C. neoformans*, resulted in proinflammatory cytokine secretion. Once the BMMs were activated, chitin was digested by secreted mammalian chitinases and subsequently phagocytosed by the macrophage. The ingested chitin then stimulated the secretion of an immunoregulatory cytokine, IL-10, that acted to inhibit the inflammatory response (24). Our *in vitro* experiments explored the initial interaction between macrophages and fungal strains with various degrees of chitin exposure. The observed association between increased chitin exposure and enhanced secretion of the proinflammatory cytokine TNF-α is therefore consistent with the previously described immune responses to chitin *in vitro*.

Alterations specifically in cell wall chitosan levels may also play a role in the detection of the *rim101*Δ mutant. Our biochemical analysis showed that the *rim101*Δ mutant has a modest increase in levels of cell wall chitosan. *C. neoformans* is among the few prominent fungal pathogens that have significant levels of chitosan in their cell walls (17, 24). Chitosan alone has been shown to activate the inflammasome in macrophages, inducing the secretion of inflammatory cytokines such as IL-18 and IL-1β. Notably, IL-1β was one of the most highly upregulated cytokines in the mouse lungs infected with the *rim101*Δ mutant (40, 41). However, the *rim101*Δ mutant does not appear to induce IL-1β secretion *in vitro*. Furthermore, the *chs3*Δ mutation did not reduce the *in vitro* TNF-α response to the *rim101*Δ mutant. The *chs3*Δ mutation disrupts almost all chitosan production (23) and would be predicted to significantly reduce chitosan levels in the *chs3*Δ *rim101*Δ strain. As we have observed with chitin, perhaps whether chitosan exposure occurs is more important for immune recognition than its total levels. *C. neoformans* chitosan may play an important role in organizing the cell wall and shielding cells from excessive immune activation; mutants with significantly decreased chitosan levels were both avirulent and capable of inducing robust and protective immune responses in multiple mouse backgrounds (49). Similarly to the response to chitin, the response to chitosan is clearly complex and likely dependent on the precise chemical form in which it is presented to the immune cells. Further experimentation is needed to more precisely determine the role of chitosan in the immune response to the *rim101*Δ mutant.

It is likely that increased chito-oligomer exposure is not the only *rim101*Δ mutant cell surface alteration that stimulates macrophages. The *rim101*Δ mutant also has a significant capsule defect which would further expose cell surface pathogen-associated molecular patterns (PAMPs) to the immune system. There is an extensive body of research demonstrating the anti-immune recognition properties of the polysaccharide capsule, showing that it both acts as a barrier to direct detection of cell wall PAMPs and actively suppresses immune signaling pathways (7–11). While direct comparisons were difficult in our *in vitro* assays, we consistently observed an increase in TNF-α secretion by macrophages under conditions of stimulation with *cap59*Δ single and double mutants compared to WT or single *rim101*Δ mutant strains. This finding also suggests that the GXM secreted by the *rim101*Δ mutant is still able to suppress immune responses.

### Mechanisms of immune recognition of fungal cell surfaces.

The innate immune receptors responding to increased *C. neoformans* chito-oligomer exposure are still unknown; however, we demonstrated here that multiple pattern recognition receptors are likely involved. The adaptor proteins required for most TLR and CLR signaling, MyD88 and CARD9, respectively, were both required for a complete response to the *rim101*Δ *cap59*Δ strain *in vitro*. Members of the TLR and CLR families have been previously implicated in chitin sensing. MyD88 and TLR2 were shown to be required for the *in vitro* proinflammatory response to chitin (46). TLR9, an intracellular receptor, was more recently shown to detect chitin that had been phagocytosed, although this interaction induced an anti-inflammatory response (24). Mannose receptor and dectin-1 have also been implicated in fungal chitin detection (24, 37). Future studies will identify the specific receptors, including non-TLR MyD88-dependent receptors such as IL-1R, required for the macrophage recognition of the *rim101*Δ mutant.

Several known chitin-sensing host proteins were not accounted for in our *in vitro* assays. Host chitinases also play an important role in the detection and killing of invading fungal pathogens. Chitotriosidase 1 (Chit1) has been associated with the induction of the nonprotective Th2 immune response during *C. neoformans* infections (50), and the acidic mammalian chitinase (AMCase) suppresses the Th2 response to fungi and other chitin-containing organisms (43, 51). The *rim101*Δ mutant and other strains with increased chito-oligomer exposure are likely more prone to attack by Chit1 and AMCase. A recent study demonstrated that IgG and the Fcγ receptor detect purified chitin particles and, when combined with several TLR agonists, induce inflammatory cytokine production from human peripheral blood mononuclear cells (48). Further analysis will determine whether chitinases and IgG are involved in the immune response to *C. neoformans* strains with various degrees of chito-oligomer exposure.

### Characterization of the immune response to the *rim101*Δ mutant *in vivo.*

The immune response to the *rim101*Δ mutant in a murine lung infection model was characterized by an increase in the levels of many proinflammatory cytokines. Levels of certain cytokines, including several favoring Th1- and IL-17-weighted responses, were particularly elevated during this infection. This response is consistent with previous studies demonstrating a strong Th1 and Th17 response to chitin or to fungal mutants producing high levels of chitin (44–46). While levels of several Th2 cytokines were elevated during the entire course of the *rim101*Δ mutant infection, IL-4 was the only cytokine whose level was significantly decreased during the *rim101*Δ mutant infection at day 14 postinfection. IL-4 secretion is an important driver of the Th2 response, as it induces the production of other Th2-associated cytokines while suppressing Th1 and Th17 responses (52). This observation demonstrates that alteration of the *rim101*Δ cell wall primarily induced a proinflammatory Th1/Th17 immune response in this infection model and is largely consistent with our previous analysis of the *rim101Δ* mutant infection (4). Here, however, we analyzed the response at later time points, and in a different mouse background (BALB/c versus C57B/6), demonstrating a consistent proinflammatory response to the *rim101*Δ mutant throughout the infection that was independent of the mouse genotype.

Many studies have demonstrated that a strong Th1 immune response is essential for clearing *C. neoformans* infections and that a Th2 response is associated with a worse disease outcome (53). However, the inflammatory condition IRIS results primarily from an overexuberant Th1 immune response (3). Our findings, in combination with findings revealing the extensive fungal cell death that occurs under this condition, suggest that increased chito-oligomer exposure may drive, or worsen, the inflammatory response in *Cryptococcus*-associated IRIS. *C. neoformans* cells have a wide range of morphologies, sizes, and cell surface properties during infection. These include the production of Titan cells, which have recently been shown to possess increased chitin levels compared to smaller yeast forms (50). In addition, dead or dying fungal cells often have aberrant cell wall compositions and arrangements (37). The results determined with the chitin synthase mutants analyzed in this study demonstrated that mutations disrupting cell wall composition can increase chito-oligomer exposure, even when these mutations are expected to decrease total chitin/chitosan levels (23). *C. neoformans* cells with altered cell wall architecture may be present in patients with recurrent or latent *C. neoformans* infections, who are particularly susceptible to developing IRIS. We previously found that the *rim101*Δ *cap59*Δ double mutant was also avirulent and cleared from infected mice, despite being highly immunostimulatory *in vitro* (4). In addition, the *chs3*Δ mutant, shown here to have increased chito-oligomer exposure, is more readily recognized by BMMs and yet is avirulent in mice (12). These examples demonstrate that the particular immune response to various levels of fungal chito-oligomer exposure can be both beneficial and detrimental to the host. Additional phenotypes, such as temperature sensitivity, would affect the fitness of certain strains during an infection. Therefore, the duration of fungal cell persistence is likely a contributor whose importance is equal to that of the cell wall organization with respect to the ultimate outcome of the infection.

### Conclusion.

We have demonstrated that the combination of increased chito-oligomer exposure and decreased capsule attachment in *C. neoformans* is strongly associated with immune stimulation *in vitro* and *in vivo*. Future experiments are needed to determine whether immune cells directly detect chitin and/or chitosan exposure or if these molecules serve as a binding site for unknown immune stimulatory molecules. Furthermore, while the *rim101*Δ mutant would rarely be encountered in the environment, understanding *C. neoformans* cell surface patterns that stimulate immune recognition is vitally important for vaccine development and prevention of damaging inflammatory responses. These data also increase our understanding of how *C. neoformans* utilizes Rim pathway signaling to actively avoid immune recognition.

## MATERIALS AND METHODS

### Strains, media, and growth conditions.

The *C. neoformans* strains used in this study are listed in Table 1. All strains were generated in the *C. neoformans* var. *grubii* strain H99 and were maintained on YPD medium (yeast extract [2%], peptone [1%], dextrose [2%]). Unless otherwise stated, *C. neoformans* strains were cultured in CO_2_-independent medium (Gibco) at 37°C for cell wall analysis and cell wall staining and prior to immune cell coculture experiments.

**TABLE 1:** Strains used in this study

*C. neoformans* strain	Genotype	Reference or source
TOC35	*rim101*Δ::*NAT*	19
KS182	*rim101*Δ::NAT + RIM101	This study
KMP18	*chs3*Δ::*NEO*	This study
KS239	*chs3*Δ::*NEO rim101*Δ::*NAT*	This study
*chs5*Δ	*chs5*Δ::*NEO*	23
KS241	*chs5*Δ::*NEO rim101*::NAT	This study
*cap59*Δ	*cap59*Δ::*NEO*	4
TOC39	*cap59*Δ::*NEO rim101*Δ::*NAT*	4

### Molecular biology.

Gene disruptions were made as previously described (54, 55) using nourseothricin (NAT) or neomycin (NEO) genes as the dominant drug resistance genes. Disruption cassettes were transformed using biolistic transformation (55). All disruptions were confirmed by Southern blotting.

### Cell wall isolation and HPLC.

*C. neoformans* strains were inoculated from an overnight YPD culture into 25 ml CO_2_-independent medium and incubated for 18 h at 37°C. Cell wall isolation and high-performance anion-exchange chromatography with pulsed amperometric detection (HPAEC-PAD) analysis were performed as previously described (56).

A modified MBTH (3-methyl–benzothiazolinone hydrazone hydrochloride) method was used to quantify chitin and chitosan levels (Jennifer Lodge, personal communication) (57, 58). Strains were cultured overnight in 50 ml YPD at 30°C and diluted to an optical density at 600 nm (OD_600_) of 0.5 in 25 ml of CO_2_-independent media. Each strain was cultured in triplicate for 18 to 20 h at 37°C with shaking. In preweighed tubes, cells were harvested, washed with H_2_O, freeze-dried, and weighed. Dried cells were then resuspended in 10 ml 6% KOH and incubated in an 80°C water bath for 90 min. Cells were spun and washed twice with phosphate-buffered saline (PBS) (pH 7.0) and then twice with H_2_O. Samples were then resuspended in H_2_O to a concentration of 10 mg/ml (using the dried cell weight) and sonicated to homogenize the samples. For each sample, two 2-ml tubes were prepared (one for chitin plus chitosan and one for chitosan only) with 100 µl (1 mg dry weight material) of the cell suspension mixed with 100 µl of 1 M HCl and subjected to vortex mixing. Two sets of hexosamine standards (d-glucosamine) were also prepared that consisted of 6 2-fold serial dilutions in 100 µl of H_2_O starting from 5 µM. One hundred microliters of 1 M HCl was also added to each standard sample. The chitin-plus-chitosan tubes were incubated for 2 h at 110°C, while the chitosan tubes were left at room temperature (RT). Heating in HCl deacetylates the chitin to chitosan, allowing total chitin/chitosan levels to be quantified. Samples were then cooled to room temperature. Next, all samples (boiled and unboiled) were deaminated as follows: in a fume hood, 400 µl of 2.5% sodium nitrite was then added to both sets of tubes, and the tubes were subjected to vortex mixing and left at room temperature for 15 min. A 200-µl volume of 12.5% ammonium sulfamate was slowly added, and the mixture was subjected to vortex mixing and incubated at room temperature for 5 min. Nitrogen oxide fumes (brown fumes) were allowed to dissipate before being removed from the fume hood. To initiate MBTH complex formation, 200 µl 0.25% MBTH (0.25% [wt/vol] 3-methyl–2-benzothiazolinone hydrazone hydrochloride monohydrate [Sigma]) was added to each sample, and the samples were subjected to vortex mixing and incubated for 30 min at 37°C. Finally, for blue color formation, 200 µl of 0.5% ferric chloride was added and the samples were mixed by pipetting and incubated for 5 min at 37°C. The samples were cooled to room temperature and spun in a microcentrifuge at maximum speed for 2 min. A 200-µl volume of the supernatant was added to a 96-well plate, and absorbance was measured at 650 nm with a spectrophotometer blanked with MBTH alone. Two standard curves corresponding to the level of absorbance versus that of hexosamine (in nanomoles) (average values) were prepared: one for chitin plus chitosan (boiled standards) and one for chitosan only (unboiled standards). Chitin levels were determined by finding the difference between chitin plus chitosan and chitosan for each sample.

### Cell wall staining and microscopy.

Cells from overnight cultures in either CO_2_-independent medium or YPD were pelleted, washed, and resuspended in phosphate-buffered saline (pH 7.4). Cells were stained either with 100 µg/ml WGA conjugated to Alexa Fluor 488 (Molecular Probes) for 45 min in the dark or with 25 µg/ml CFW for 10 min. Cells were washed 2 times with PBS and imaged on a Zeiss Axio Imager A1 fluorescence microscope equipped with an AxioCam MRm digital camera. The same exposure time was used to image all strains. The mean gray value (sum of gray values for all the pixels in a cell divided by the number of pixels that make up the cell) was calculated for at least 100 individual cells using ImageJ (Fiji) (59), and the results are reported as average fluorescence values ± the standard errors of the means.

### *In vitro* macrophage experiments.

Bone marrow-derived macrophages were isolated and prepared as previously described (60, 61). The harvested cells were plated in 96-well plates in Dulbecco’s modified Eagle’s medium (DMEM) with 10% fetal bovine serum (FBS) (non-heat inactivated) and 1 U/ml penicillin/streptomycin at a concentration of 5 × 10^4^ cells/well. A total of 5 × 10^5^ *C. neoformans* cells (10:1 *C. neoformans* cells/macrophages) were added to each well, and the coculture was incubated at 37°C and 5% CO_2_. Each fungal strain was tested with a minimum of three biological replicates per experiment. After 6 h of coincubation, the medium from these experiments was harvested, and secreted TNF-α was quantified by enzyme-linked immunosorbent assay (ELISA; BioLegend). Data are represented as the average TNF-α values (in picograms per milliliter) for these biological triplicates or as these values normalized to macrophage-only control wells in which fresh tissue culture medium alone, rather than fungal cells, was added to each well. This type of normalization allowed comparisons between experiments performed on different days with different macrophage cultures and different ELISA plates.

All strains with the *cap59*Δ mutation aggregated in large, adherent clumps that made accurate quantification by hemocytometer or optical density impossible. Instead, these were normalized to 2 mg wet cell pellet/ml of medium. This cell concentration was used since it approximated the milligram-per-milliliter concentration of the *rim101*Δ mutant inoculum for the standard multiplicity of infection (MOI) of 10 fungal cells to 1 BMM used for the other coculture assays performed in this study (the *rim101*Δ strain was used as a comparator because it has a significant capsule defect and therefore has a mass/cell ratio similar to that of the acapsular *cap59*Δ strains). A concentration of 2 milligrams/ml of the *rim101*Δ mutant was approximately 5 × 10^6^ cells/ml.

Cell separation experiments were performed using Transwell (Corning) tissue culture plates. Bone marrow-derived macrophages were harvested and plated (as described above) in the lower Transwell chamber. The various *C. neoformans* strains were incubated in the upper chamber and separated from the macrophages by a 3-µm-pore-size membrane to prevent cell migration while allowing free transit of diffusible molecules between the two chambers.

To test the effect of isolated cell walls on macrophage activation, cell wall material (isolated as described above) was added at a concentration of 10 mg/ml to BMMs and incubated at 37°C and 5% CO_2_ for 6 h. After coincubation, the medium from these experiments was harvested, and secreted TNF-α was quantified by ELISA as described above.

### Animal experiments.

We used the murine inhalation model of cryptococcosis to assess organ fungal burden. BALB/c mice were intranasally infected with 10^4^ fungal cells for each strain. At specified time points, the animals were sacrificed, and lungs and brains were dissected and homogenized in 2 ml PBS. Viable cells were calculated as CFU counts per gram of tissue by quantitative culture. Pulmonary leukocyte isolation, staining, and flow cytometry were assessed as described previously (62). Lung cytokine levels were quantified from lung homogenates as described previously (62). All animal experimentation was performed in accordance with institutional guidelines established at Duke University and the University of Texas at San Antonio.
