# Supplementary material for: Rim Pathway-Mediated Alterations in the Fungal Cell Wall Influence Immune Recognition and Inflammation
Source: mBio. 2017 Jan 31;8(1):e02290-16. doi: 10.1128/mBio.02290-16 (PMC5285508; doi:10.1128/mBio.02290-16)
Supplement: TABLE S1 [file mbo001173174st1.docx]

Supplementary Table 1: TNF-α pg/ml for Figure 1

|  |  | **Mean (pg/ml TNF-α)** | **SEM** | **N** |
| --- | --- | --- | --- | --- |
| Live | WT | 5.79 | 1.32 | 18 |
|  | *rim101*Δ | 90.77 | 17.35 | 9 |
|  | *rim101*Δ*+RIM101* | 9.61 | 1.026 | 9 |
| Heat-Killed | WT | 13.18951 | 3.30 | 18 |
|  | *rim101*Δ | 117.6363 | 15.98 | 9 |
|  | *rim101*Δ*+RIM101* | 18.80357 | 4.10 | 9 |
|  | No Cn Control | 1.43 | 0.28 | 18 |
