# Supplementary material for: Rim Pathway-Mediated Alterations in the Fungal Cell Wall Influence Immune Recognition and Inflammation
Source: mBio. 2017 Jan 31;8(1):e02290-16. doi: 10.1128/mBio.02290-16 (PMC5285508; doi:10.1128/mBio.02290-16)
Supplement: TABLE S2 [file mbo001173174st2.docx]

Supplementary Table 2: TNF-α pg/ml for Figure 2C. BMM TNF-α response toward *C. neoformans* cultured in rich media vs. tissue culture media

|  |  | **Mean (pg/ml TNF-α)** | **SEM** | **N** |
| --- | --- | --- | --- | --- |
| Rich Media | WT | 1.92 | 0.20 | 8 |
|  | *rim101*Δ | 2.26 | 0.30 | 8 |
| TC media | WT | 3.36 | 0.23 | 8 |
|  | *rim101*Δ | 11.84 | 1.39 | 8 |
|  | No Cn Control | 2.50 | 0.19 | 16 |
