# Supplementary material for: Rim Pathway-Mediated Alterations in the Fungal Cell Wall Influence Immune Recognition and Inflammation
Source: mBio. 2017 Jan 31;8(1):e02290-16. doi: 10.1128/mBio.02290-16 (PMC5285508; doi:10.1128/mBio.02290-16)
Supplement: TABLE S3 [file mbo001173174st3.docx]

**Supplementary Table 3: All cytokines quantified**

|  |  | **WT** | | ***rim101*Δ** | | ***rim101*Δ *+ RIM101*** | |
| --- | --- | --- | --- | --- | --- | --- | --- |
|  |  | Mean | SEM | Mean | SEM | Mean | SEM |
| **IL-1α** | **Day 7** | 135.72 | 16.48 | 285.27 | 65.56 | 113.72 | 18.54 |
|  | **Day 14** | 312.18 | 56.38 | 1493.17 | 403.76 | 283.14 | 57.05 |
| **IL-1β** | **Day 7** | 579.83 | 90.71 | 930.61 | 164.37 | 466.70 | 83.81 |
|  | **Day 14** | 1970.42 | 325.55 | 4643.47 | 867.83 | 1562.72 | 174.80 |
| **IL-2** | **Day 7** | 25.91 | 2.96 | 53.04 | 10.76 | 20.84 | 2.76 |
|  | **Day 14** | 20.40 | 3.64 | 25.20 | 1.87 | 20.55 | 3.05 |
| **IL-3** | **Day 7** | 10.70 | 1.06 | 16.97 | 2.41 | 9.20 | 1.16 |
|  | **Day 14** | 10.93 | 0.62 | 21.81 | 3.69 | 9.45 | 0.40 |
| **IL-4** | **Day 7** | 32.47 | 3.92 | 100.65 | 14.48 | 28.21 | 3.50 |
|  | **Day 14** | 253.73 | 51.94 | 90.66 | 27.41 | 292.22 | 36.36 |
| **IL-5** | **Day 7** | 11.90 | 1.55 | 27.08 | 2.85 | 13.18 | 1.94 |
|  | **Day 14** | 16.79 | 3.24 | 26.54 | 8.08 | 36.95 | 8.15 |
| **IL-6** | **Day 7** | 77.04 | 11.16 | 112.92 | 27.05 | 76.27 | 12.32 |
|  | **Day 14** | 51.76 | 5.26 | 174.70 | 42.24 | 51.22 | 7.16 |
| **IL-9*** | **Day 7** | 41.34 | 9.26 | 67.13 | 12.96 | 44.38 | 3.42 |
|  | **Day 14** | 134.35 | 24.30 | 112.96 | 26.55 | 161.16 | 32.12 |
| **IL-10** | **Day 7** | 26.13 | 3.24 | 40.77 | 7.08 | 23.22 | 3.31 |
|  | **Day 14** | 45.41 | 3.60 | 78.99 | 10.63 | 47.27 | 2.79 |
| **IL-12(p40)** | **Day 7** | 87.97 | 8.54 | 78.76 | 13.06 | 72.85 | 11.55 |
|  | **Day 14** | 125.63 | 19.10 | 300.75 | 75.04 | 134.23 | 11.55 |
| **IL-12(p70)** | **Day 7** | 106.28 | 18.57 | 155.24 | 35.42 | 98.87 | 20.26 |
|  | **Day 14** | 138.46 | 13.22 | 386.01 | 82.02 | 137.60 | 15.07 |
| **IL-13** | **Day 7** | 121.13 | 9.53 | 182.82 | 19.17 | 109.21 | 9.71 |
|  | **Day 14** | 340.78 | 57.72 | 368.41 | 57.69 | 497.64 | 115.09 |
| **IL-17** | **Day 7** | 19.29 | 2.69 | 34.71 | 5.48 | 17.38 | 4.49 |
|  | **Day 14** | 24.13 | 4.95 | 34.94 | 5.12 | 18.13 | 2.87 |
| **Eotaxin*** | **Day 7** | 308.44 | 35.75 | 361.46 | 33.17 | 265.93 | 28.39 |
|  | **Day 14** | 415.69 | 19.44 | 496.75 | 27.71 | 430.69 | 22.58 |
| **G-CSF** | **Day 7** | 182.24 | 24.49 | 312.63 | 39.71 | 160.15 | 25.98 |
|  | **Day 14** | 100.67 | 16.24 | 639.01 | 180.14 | 80.26 | 9.44 |
| **GM-CSF** | **Day 7** | 73.69 | 15.68 | 100.47 | 21.00 | 74.93 | 19.11 |
|  | **Day 14** | 72.07 | 15.45 | 93.50 | 20.20 | 71.96 | 15.84 |
| **IFN-γ** | **Day 7** | 12.07 | 0.94 | 17.71 | 3.07 | 9.90 | 1.07 |
|  | **Day 14** | 13.00 | 0.70 | 23.60 | 2.95 | 12.90 | 0.39 |
| **CXCL1** | **Day 7** | 571.29 | 54.74 | 861.03 | 152.09 | 467.69 | 65.49 |
|  | **Day 14** | 479.10 | 48.50 | 1283.06 | 236.07 | 524.93 | 54.73 |

*** Cytokines not displayed in Figure 6.**
